# The Charlson Comorbidity Index and depression are associated with satisfaction after short-segment lumbar fusion in patients 75 years and older

**DOI:** 10.3389/fsurg.2022.991271

**Published:** 2022-09-12

**Authors:** Shuai-Kang Wang, Hong Mu, Peng Wang, Xiang-Yu Li, Chao Kong, Jing-bo Cheng, Shi-Bao Lu, Guo-Guang Zhao

**Affiliations:** ^1^ Department of Orthopedics, Xuanwu Hospital, Capital Medical University, Beijing, China; ^2^ National Clinical Research Center for Geriatric Diseases, Xuanwu Hospital, Capital Medical University, Beijing, China

**Keywords:** elderly, lumbar fusion surgery, comprehensive geriatric assessment, dissatisfaction, depression, comorbiditiy

## Abstract

**Background:**

The rate and volume of lumbar spinal fusion (LSF) surgery performed for patients aged 75 years and older increased in recent years. The purposes of our study were to identify factors associated with postoperative dissatisfaction and evaluate the predictive value of comprehensive geriatric assessment (CGA) for dissatisfaction at 2 years after elective short-segment (one- or two- level) LSF in patients aged 75 and older.

**Methods:**

This was a retrospective study using a prospectively collected database of consecutive patients (aged 75 and older) who underwent elective short-segment transforaminal lumbar interbody fusion surgery for degenerative diseases from June 2018 to May 2020. Preoperative CGA consisting six domains was performed for each patient 1 day before the operative day. Univariate and multivariate analyses were performed to identify factors that predict for dissatisfaction with surgical treatment. The primary outcome was patient satisfaction with LSF surgery, as measured by the North American Spine Society (NASS) satisfaction scale. Secondary outcomes included postoperative complications, the length of stay, visual analog scale (VAS), and Oswestry Disability Index.

**Results:**

A total of 211 patients were available for a follow-up at 2 years and included in our final study cohort with a mean age of 80.0 years. A total of 175 patients (82.9%) were included in the satisfied group, and 36 patients (17.1%) were included in the not dissatisfied group. In the dissatisfied group, there was a higher incidence of postoperative complications (30.6% vs. 14.3%, *p* = 0.024) and greater VAS scores for lower back (4.3 ± 1.9 vs. 1.3 ± 1.4, *p* = 0.001) and leg (3.9 ± 2.1 vs. 0.9 ± 1.3, *p* = 0.001). Multivariate regression analysis revealed that patients with greater CCI score [odd ratio (OR) 2.56, 95% CI, 1.12–5.76; *p* = 0.030 for CCI 1 or 2 and OR 6.20, 95% CI, 1.20–28.69; *p* = 0.024], and depression (OR 3.34, 95% CI, 1.26–9.20; *p* = 0.016) were more likely to be dissatisfied compared with patients with the CCI score of 0 and without depression.

**Conclusions:**

Satisfaction after LSF in older patients (aged 75 and older) was similar to that of previously reported younger patients. Preoperative depression and higher CCI scores were independent risk factors for postoperative dissatisfaction two years after LSF surgery. These results help inform decision-making when considering LSF surgery for patients aged 75 and older.

## Introduction

With the rapid population aging, the incidence of lumbar degenerative disease is increasing, severely deteriorating the patient's quality of life (QoL), and increasing socioeconomic burdens (1). Lumbar spinal fusion (LSF) surgery is the standard treatment for lumbar degenerative disease. With the improvement of surgical techniques, the rate and volume of LSF surgery increased in recent years. Martin et al. (2) reviewed the National Inpatient Sample database and found that aggregate hospital costs increased by 177%, and the volume of elective lumbar fusion increased 62.3% from 2004 to 2015 in the United States, especially for elderly patients. A recent study using Finnish nationwide data showed that the increase in lumbar spinal fusions was highest among women over 75 years, with a 4-fold increase (3). Previous studies found that age did not impact on patient-reported outcomes (4–6). However, patients aged 75 and older may be more likely to refuse surgery than younger patients due to fear of high rates of morbidity and mortality. With the increase of age, the physiological function reserves of elderly patients decrease, especially changes in cardiopulmonary function and the nervous system (7). These may lead to reduced ability of older patients to tolerate surgical stress. Comprehensive assessments are needed to select patients who are more likely to benefit from surgical treatment when designing the treatment plans for high-risk patients.

Patient-reported outcome (PRO) measures of pain, disability, and health-related QoL are commonly used to evaluate the effect of LSF. Sivaganesan et al. (8) reported that 23% of patients with clinically relevant pain improvement nevertheless remained dissatisfied with surgery. Patient satisfaction is essential to evaluate the quality and effectiveness of medical care. Reimbursement of healthcare systems is linked to patient-reported satisfaction in many countries. Improving postoperative satisfaction is necessary for the current healthcare environment. Patient characteristics such as smoking status, psychological distress, low level of education, unemployment status and symptoms duration were associated with postoperative dissatisfaction in previous studies (9–13). The comprehensive geriatric assessment (CGA) is an effective tool for assessing a patient's functional age. The CGA evaluates an elderly patient's medical, psychosocial, functional, and environmental resources and links them with an overall plan of treatment and follow-up. Preoperative assessment can identify patients with physiologic dysfunction, including frailty, disability, depression, and malnutrition associated with poor clinical outcomes (14). The value of CGA in predicting long-term QoL and satisfaction has been demonstrated in previous studies on cancer surgery and hip fracture surgery (15–17); however, few studies on spine surgery included CGA in their analyses (18). Identifying the factors influencing older patient satisfaction after LSF surgery is critical to improve shared clinical decision-making. Thus, this study aimed to determine the level of satisfaction and identify factors associated with postoperative dissatisfaction 2 years after elective short-segment (one- or two- level) LSF in patients aged 75 and older.

## Materials and methods

This was a retrospective study using a prospectively collected database. The ethical review committee of our hospital approved the study. Lumbar spine surgery is recommended for patients who have failed conservative treatment with medications or exercise for more than half a year, and the same experienced surgical team performed all surgeries. A midline incision was made for patients under general anesthesia. The nerve roots were decompressed by hemilaminectomy or laminectomy according to the preoperative lumbar symptoms, radicular symptoms and MRI. The vertebral pedicle screws of surgical segments were implanted under direct vision. To improve the fusion rates and restore the height of intervertebral height, cages filled with bone grafts were placed in the intervertebral space. We included consecutive patients (aged 75 and older) who underwent elective short-segment transforaminal lumbar interbody fusion surgery for degenerative diseases from June 2018 to May 2020. Patients undergoing surgery for lumbar trauma, tumors, infections were excluded.

### Data collection

As published previously, we collected patients' demographic variables [age, sex, weight, body mass index (BMI), comorbidities, smoking status, and surgical history], American Society of Anesthesiologists (ASA), laboratory examination data (level of albumin, prealbumin and hemoglobin), primary diagnosis and baseline PRO scores [visual analogue scale (VAS), Oswestry Disability Index (ODI)], and surgery-related variables (operative time, number of fused levels, and estimated blood loss).

We conducted preoperative CGA for each patient 1day before the operative day. Our CGA consisted of six domains [Zung Depression Rating Scale (ZDRS), Activities of Daily Living (ADL), Instrumental Activities of Daily Life (IADL), Mini-Mental Status Examination (MMSE), Mini Nutritional Assessment (MNA), and Charlson Comorbidity Index (CCI)]. The severity of comorbidities was evaluated using CCI (19) and ASA grades. Functional status and dependency were evaluated using ADL (20) and IADL (21) scales. Cognitive function and psychological disorders were evaluated using the MMSE (22) and ZDRS (23, 24), respectively. The six domains of our CGA with their corresponding cutoff values are summarized in Table 1.

**Table 1:** Cutoff values for the six domains of our comprehensive geriatric assessment.

Domains	Cutoff values for a deficit or disability
CCI	No applicable
ADL	Independent: 100 points; mild disability: 61–99 points; severe disability: ≤60 points
IADL	Independent: 100 points; mild disability: 61–99 points; severe disability: ≤60 points
MNA	Malnutrition: ≤18 points
MMSE	Cognitive impairment: ≤23 points
Zung depression	Depression: ≥50 points

CCI, Charlson Comorbidity Index; ADL, Activities of Daily Living; IADL, Instrumental Activities of Daily Living; MNA, Mini Nutritional Assessment; MMSE, Mini-Mental Status Examination.

### Outcome measures

The primary outcome was patient satisfaction with LSF surgery at a 2-year follow-up, measured by the North American Spine Society (NASS) satisfaction scale. Answer choices of the satisfaction scale were as follows: (1) The treatment met my expectations; (2) I did not improve as much as I had hoped, but I would undergo the same treatment for the same outcome; (3) I did not improve as much as I had hoped, and I would not undergo the same treatment for the same outcome; and (4) I am the same or worse than before treatment. Patients who chose (1) and (2) were considered satisfied with surgical care and outcomes, and patients with the other answer choices were classified as dissatisfied and regretting the choice of surgical treatment (12). Secondary outcomes included VAS scores of lower back and leg pain, ODI, the incidence of complications, and the length of hospital stay.

### Statistical analysis

Continuous variables were expressed as mean ± standard deviation and analyzed using the 2-tailed Student's *t*-test or Mann-Whitney *U* test depending on the variable type. Categorical variables were expressed as frequencies with percentages and analyzed using Fisher's exact or chi-square tests. All variables with a *p*-value <0.1 detected in univariate analyses were entered into multivariate logistic analyses for dissatisfaction. All statistical analyses were performed using SPSS Statistics 25 (SPSS, version 22.0, Inc., Chicago, IL, USA). Statistical significance was set at *p* < 0.05.

## Results

**Figure 1:**
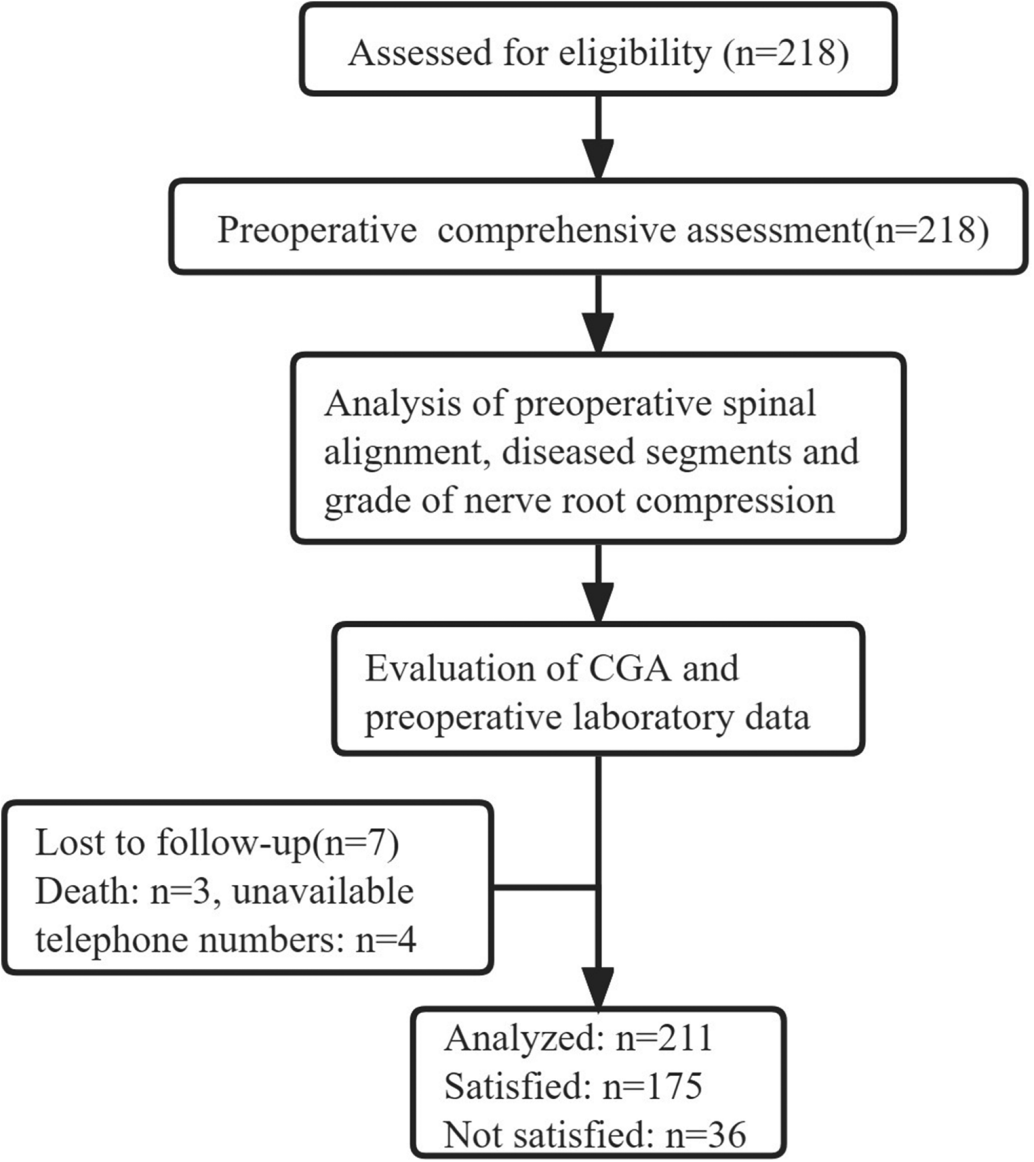
Flow chart of study participants.

**Figure 2:**
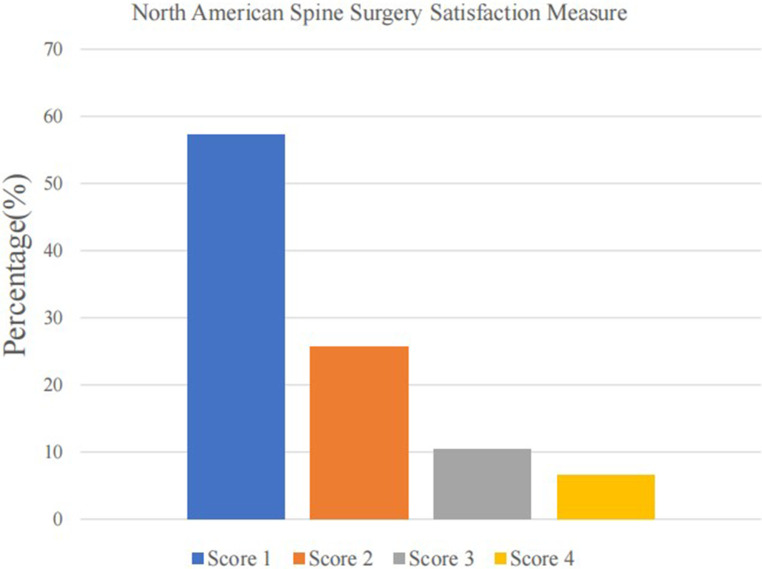
North American Spine Surgery Satisfaction measure.

A total of 218 consecutive patients (75 years or older) underwent short-segment LSF surgery for lumbar degenerative diseases from June 2018 to May 2020. All patients received preoperative assessments by a multidisciplinary team including experienced surgeons, internists, and anesthesiologists. Of the included 218 patients, three patients died of other diseases after the patient had been discharged home and four patients were lost to follow-up. A total of 211 patients were available for a follow-up at 2 years and included in our final study cohort with a mean age of 80.0. (Figure 1). Among these, 121 (57.3%) patients had a NASS satisfaction score of 1 at 2-year follow-up, 54 (25.6%) patients had a score of 2, 22 (10.4%) patients had a score of 3, and 14 (6.6%) patients had a score of 4 (Figure 2). A total of 175 patients (82.9%) were satisfied after 2 years of LSF surgery and included in the satisfied group, and 36 (17.1%) were dissatisfied and included in the not dissatisfied group. There were no significant differences between groups in baseline demographic characteristics, primary diagnosis, comorbidities, or laboratory data (Table 2). Compared to satisfied patients, dissatisfied patients showed a higher incidence of depression (25.0% vs. 9.1%, *p *= 0.002) and a higher rate of greater CCI scores (*p *= 0.017). There were no significant differences in ASA level (*p *= 0.383), ADL (*p *= 0.631), IADL (*p *= 0.682), MNA (*p *= 0.910), MMSE (*p* = 0.132), or procedure-related variables (Table 3). Table 4 presents the study population's postoperative clinical outcomes and VAS scores. In the dissatisfied group, there was a higher incidence of postoperative complications (30.6% vs. 14.3%, *p* = 0.024), greater ODI (44.0 ± 26.3 vs. 20.3 ± 17.2, *p* = 0.001) and VAS scores for lower back (4.3 ± 1.9 vs. 1.3 ± 1.4, *p *= 0.001) and leg (3.9 ± 2.1 vs. 0.9 ± 1.3, *p *= 0.001). There were no differences between the two groups in postoperative deep vein thrombosis, surgical site infection, and urinary retention.

**Table 2:** Baseline characteristics and laboratory data of patients in the two groups.

Variable	Total (*n* = 211)	Satisfied (*n* = 175)	Not Satisfied (*n* = 36)	*p*-value
Female *n*/ (%)	126 (59.7%)	108 (61.7%)	18 (50.0%)	0.192
Age (year)	80.0 ± 3.5	79.9 ± 3.5	80.0 ± 3.4	0.935
Height (cm)	160.8 ± 7.5	160.6 ± 7.5	162.0 ± 7.5	0.295
Weight (kg)	63.9 ± 9.2	64.0 ± 9.2	63.5 ± 8.9	0.774
BMI (kg/m^2^)	24.8 ± 3.6	24.9 ± 3.7	24.2 ± 3.1	0.311
Co-Morbidities n/ (%)				
Hypertension	140 (66.4%)	117 (66.9%)	23 (63.9%)	0.731
Coronary heart disease	52 (24.6%)	42 (24.0%)	10 (27.8%)	0.632
Diabetes disease	53 (25.1%)	41 (23.4%)	12 (33.3%)	0.212
Knee arthritis	27 (12.8%)	22 (12.6%)	5 (13.9%)	0.829
Digestive disease	26 (12.3%)	20 (11.4%)	6 (16.7%)	0.384
Old cerebral infarction	14 (6.6%)	11 (6.3%)	3 (8.3%)	0.653
Pulmonary disease	3 (1.4%)	2 (1.1%)	1 (2.8%)	0.450
Osteoporosis	44 (20.9%)	35 (20%)	9 (25%)	0.501
Urological diseases	18 (8.5%)	15 (8.6%)	3 (8.3%)	0.963
Smoker	14 (6.6%)	12 (6.9%)	2 (5.6%)	0.775
Drinker	15 (7.1%)	13 (7.4%)	2 (5.6%)	0.435
Diagnosis				0.151
LSS	77 (36.5%)	60 (34.3%)	17 (47.2%)	
DDD	89 (37.9%)	79 (45.1%)	10 (27.8%)	
Lumbar	45 (21.3%)	36 (20.6%)	9 (25.0%)	
Spondylolisthesis				
Duration of symptoms (year)	6.4 ± 9.2	6.2 ± 9.5	6.9 ± 7.4	0.629
VAS (lower back)	5.1 ± 2.0	5.1 ± 2.1	5.2 ± 1.6	0.828
VAS (leg)	6.7 ± 2.3	6.7 ± 2.3	6.4 ± 2.3	0.411
ODI	54.5 ± 12.4	54.6 ± 12.7	53.9 ± 11.4	0.781
Laboratory data				
Serum albumin (g/L)	37.5 ± 3.8	37.7 ± 3.8	36.5 ± 3.6	0.075
Prealbumin (g/L)	219 ± 55	219 ± 55	218 ± 57	0.881
Hemoglobin (g/L)	126 ± 15	127 ± 14	123 ± 17	0.119

BMI, Body Mass Index; LSS, lumbar spine stenosis; DDD, Degenerative Disc Disease; VAS, Visual Analogue Scale; ODI, Oswestry Dability Index.

**Table 3:** CGA scores and procedure-related variables of patients in the two groups.

Variable	Satisfied (*n* = 175)	Not Satisfied (n = 36)	*p*-value
ASA			0.383
1 or 2	77 (44.0%)	13 (36.1%)	
3 or 4	98 (56.0%)	23 (63.9%)	
CCI			**0**.**017**
0 (%)	96 (54.9%)	11 (30.5%)	** **
1 or 2	74 (42.3%)	22 (61.1%)	** **
3 and 3+	5 (2.8%)	3 (8.3%)	** **
Level of dependence in the ADL			0.631
Independent	31 (17.7%)	7 (19.4%)	
Mild disability	102 (58.3%)	18 (50.0%)	
Severe disability	42 (24.0%)	11 (30.6%)	
Level of dependence in the IADL			0.682
Independent	39 (22.2%)	6 (16.7%)	
Mild disability	53 (30.3%)	13 (36.1%)	
Severe disability	83 (47.4%)	17 (47.2%)	
Zung depression scale			**0**.**002**
Depression	16 (9.1%)	9 (25%)	
No depression	159 (90.9%)	27 (75%)	
MNA			
Malnutrition	47 (26.9%)	10 (27.8%)	0.910
No malnutrition	128 (73.1%)	26 (72.2%)	
MMSE			0.132
Cognitive impairment	42 (24.0%)	13 (36.1%)	
Normal cognition	133 (76.0%)	23 (63.9%)	
No. of levels			0.923
1	50 (28.6%)	10 (27.8%)	
2	125 (71.4%)	26 (72.2%)	
Operative time (min)	199.7 ± 61.4	212.9 ± 56.1	0.237
EBL	334.1 ± 260.0	390.1 ± 261.0	0.234

ASA, American Society of Anesthesiologists Physical Classification System; CCI, Charlson Comorbidity Index; ADL, Activities of Daily Living; IADL, Instrumental Activities of Daily Living; MNA, Mini Nutritional Assessment; MMSE, Mini-Mental Status Examination; EBL, Estimated Blood Loss.

Bold values implies statistical significance.

**Table 4:** Postoperative outcomes of patients in both groups.

Variables	Satisfied (n = 175)	Not Satisfied (n = 36)	*p-*value
*NASS Satisfaction Measure*	1.3** **±** **0.5	3.4** **±** **0.5	**0**.**001**
VAS of lower back	1.3** **±** **1.4	4.3** **±** **1.9	**0**.**001**
VAS of leg	0.9** **±** **1.3	3.9** **±** **2.1	**0**.**001**
Length of hospital stay	17.2** **±** **7.5	18.3** **±** **6.6	0.413
Complications	25 (14.3%)	11 (30.6%)	**0**.**024**
Urinary retention	4 (2.3%)	2 (5.6%)	0.282
Deep vein thrombosis	2 (1.1%)	2 (1.1%)	0.077
Nausea/vomiting	9 (5.1%)	4 (11.1%)	0.175
Urinary Infection	1 (0.6%)	0 (0%)	0.649
Acute cerebral infarction	1 (0.6%)	0 (0%)	0.649
Pneumonia	2 (1.1%)	1 (2.8%)	0.450
Hematoma	2 (1.1%)	0 (0%)	0.519
Delirium	2 (1.1%)	2 (5.5%)	0.077
Myocardial infarction	1 (0.6%)	1 (2.8%)	0.213
Surgical site infection	3 (1.7%)	1 (2.8%)	0.670
Constipation	3 (1.7%)	2 (5.5%)	0.168

NASS, North American Spine Surgery; VAS, Visual Analogue Scale.

Bold values implies statistical significance.

**Figure 3:**
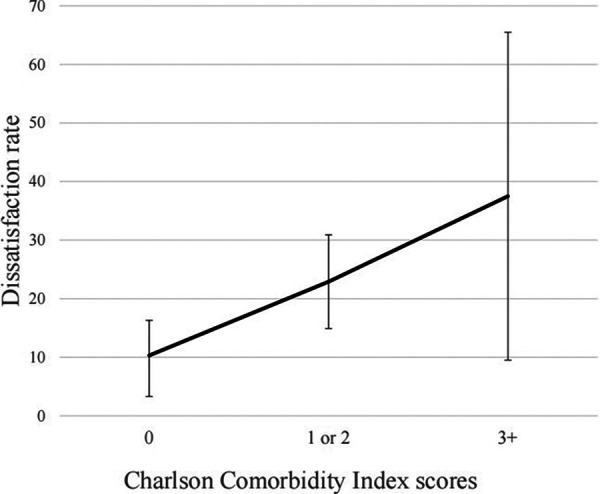
Association between CCI and dissatisfaction rate.

Five factors (CCI score, depression, preoperative serum albumin, complications) with a *p*-value <0.1 in univariate analyses were included in multivariate logistic analyses. Multivariate regression analysis revealed that patients with greater CCI score [odds ratio, (OR) 2.56, 95% CI, 1.12–5.76; *p *= 0.030 for CCI 1 or 2 and OR 6.20, 95% CI, 1.20–28.69; *p* = 0.024], and depression (OR 3.34, 95% CI, 1.26–9.20; *p* = 0.016) were more likely to be dissatisfied with surgical treatment compared with patients with the CCI score of 0 (Table 5). Increasing CCI score was significantly associated with a higher rate of dissatisfaction (Figure 3). However, preoperative serum albumin and postoperative complications were not significantly associated with dissatisfaction.

**Table 5:** Multivariate logistic analysis for risk factors associated with dissatisfaction.

Variable	OR	95% CI	*p-*value
Depression	3.40	1.26–9.20	**0**.**016**
CCI score of 1or 2	2.56	1.12–5.76	**0**.**030**
CCI score of 3+	6.20	1.20–28.69	**0**.**024**

CCI, Charlson Comorbidity Index; LL, Lumbar Lordosis.

Bold values implies statistical significance.

## Discussion

Patients of advanced age had a higher incidence of postoperative complications, readmission, and hospital costs after spinal surgery due to the presence of frailty and comorbidities (25–27); these findings suggest that comprehensive assessment is needed to select patients more likely to benefit from surgical treatment. Preoperative patient expectations, postoperative pain control levels, functional recovery, and cost of hospitalization are influenced by satisfaction. The NASS scale is a commonly used evaluation tool for patient-reported outcomes considering patient expectations and postoperative outcomes actuality. The objective of the present study was to measure the satisfaction of patients aged 75 years and older using the NASS scale and to identify independent risk factors for dissatisfaction with short-segment lumbar fusion surgery.

Previously, it was demonstrated that older age was not a risk factor for worse PRO, and elderly patients could also benefit from spinal fusion and show a good satisfaction rate (6, 28, 29). Our study found that 82.9% of patients were satisfied, and 6.6% were most dissatisfied with surgical treatment at the 2-year follow-up point. These results were similar to previous studies. Mummaneni et al. reviewed 502 patients (mean age of 61 years) undergoing surgery for degenerative lumbar spondylolisthesis and found that 82% of patients were satisfied with and 10.3% of patients were most dissatisfied with their surgery (12). Another study of patients over 80 years conducted by Hikata et al. found that 77.5% of patients were satisfied with surgical treatment (29). Our study validates previous findings that LSF surgery effectively improve QoL in patients aged 75 years and older.

The predictive value and details of CGA were extensively reported in various disciplines (18, 30, 31). Several studies evaluated the value of CGA and found that preoperative ASA grade, frailty, depression, and CCI scores were significantly associated with postoperative complications and PRO following spinal surgery (32, 33). The present study found that higher CCI scores and depression were independently associated with postoperative dissatisfaction. The CCI is a convenient tool that allows physicians to assess comorbidity severity and predict mortality risk for surgery patients (19). In a prospective observational study, Whitmore et al. (33) found that increasing CCI score was associated with an increased likelihood of major and minor complications. In another study of patients with single-level fusion surgery, the CCI score was a risk factor for less improvement in the Japanese Orthopedic Association lumbar score (34). Moreover, the CCI was also reported to be independently associated with length of hospital stay and unplanned readmission after lumbar spine surgery (35). Some researchers used other satisfaction evaluation tools to demonstrate the relationship between satisfaction and CCI and found the same conclusion. Benjamin et al. (36) conducted a retrospective review of 17,853 consecutive spinal patients and found that overall comorbid disease burden was a significant negative predictor for high Press Ganey satisfaction scores. Another study reported that high CCI was associated with lower Hospital Consumer Assessment of Healthcare Providers and Systems score of satisfaction (37). In the present study, we compared the baseline characteristics of the satisfied group with the dissatisfied group and found that no specific disease was associated with dissatisfaction. It is worth noting that the impact of comorbidities on satisfaction is multifaceted, and this finding highlights the importance of comprehensive assessments in patients aged 75 and older.

The ZDRS is a 20-item questionnaire with well-established reliability and validity (23). Depression, as measured using the Zung depression scale, was another domain of CGA associated with postoperative dissatisfaction in elderly patients (aged 75 years and older). The association between preoperative depression and postoperative outcomes was demonstrated in previous studies (9, 10). In a retrospective study of 8,585 patients, Zakaria et al. (38) found that preoperative depression (measured using the Patient Health Questionnaire-2) predicted worse satisfaction and inability to return to work. In another retrospective study, Levin et al. (9) analyzed the association between depression using the PHQ-9 and postoperative satisfaction after lumbar fusion. These results showed that patients with preoperative depression were more likely to be dissatisfied with physicians and nurses. The ZDSR was also identified to be effective in predicting postoperative satisfaction in patients undergoing revision lumbar surgery (39). Depressed patients (particularly those more than 75 years) may be more sensitive to preoperative mental stress and postoperative pain. It is necessary for these patients to understand their expectations and fully provide them with emotional support. Changes in depressive symptoms may have a more significant effect than preoperative depression on satisfaction and changes in other PRO after spine surgery (40).

In some previous studies, patient satisfaction showed a clear correlation with achieving clinical improvement in pain and disability after surgery (12, 29, 41). Nevertheless, Yoo et al. (42) found that actual postoperative results had a stronger correlation with patient satisfaction than the expectation-actuality discrepancy and postoperative improvement. In the present study, we found no difference in preoperative pain level and functional disability between the satisfied and unsatisfied groups, while the unsatisfied group had significantly higher postoperative pain scores. These findings suggest that surgeons should focus on achieving the best clinical outcome through surgery regardless of the duration and extent of the patient's preoperative symptoms.

Another postoperative outcome that should be noted is the incidence of postoperative complications. Consistent with several previous studies (12, 37, 43), multivariate regression analysis revealed that complication was not an independent risk factor for postoperative dissatisfaction in our patient cohort. Some reasons may potentially explain this finding. First, there were no severe postoperative complications such as myocardial infarction, cerebral infarction, or paralysis in our enrolled patients, and all patients with complications were discharged from the hospital after medical and surgical treatment. Second, the impact of confounding factors was amplified due to the small sample size. Moreover, preoperative comorbidities may have potential implications for postoperative complications, and these variables have a synergistic effect on postoperative satisfaction.

Several limitations in our study should be noted. First, this was a retrospective, single-center study evaluating the impact of CGA on satisfaction and postoperative CGA was not performed for patients. Prospective studies are needed to identify the changes in CGA score after surgery and the impact of improvement of preoperative depression on outcomes. Second, the small sample size of our study may decrease our findings' robustness. Third, only six CGA domains, pain level, and functional status were included, and QoL scales (e.g., Short Form 36 Health Status Survey, PHQ-9) that may be associated with satisfaction were evaluated. Finally, this study had a short follow-up time of 24 months. Indeed, satisfaction is an outcome that can fluctuate with the follow-up time. Long-term and continuous follow-up will help to identify changes in satisfaction over time. Despite these limitations, this is the first study to examine the value of the CGA for predicting surgical outcomes in patients aged 75 and older. Our findings could be implemented in clinical practice to improve shared decision-making when considering LSF for patients aged 75 and older.

## Conclusions

The results of this study indicate that the satisfaction after LSF in older patients (aged 75 and older) was similar to that of previously reported younger patients. Multivariate analysis revealed that preoperative depression and higher CCI scores were independent risk factors for postoperative dissatisfaction two years after LSF surgery. Preoperative assessment using the Zung depression scale and CCI help inform decision-making when considering LSF surgery for patients aged 75 and older.

## Data Availability

The original contributions presented in the study are included in the article/Supplementary Material, further inquiries can be directed to the corresponding author/s.
